# Effect of the ROCK inhibitor fasudil on the brain proteomic profile in the tau transgenic mouse model of Alzheimer's disease

**DOI:** 10.3389/fnagi.2024.1323563

**Published:** 2024-02-19

**Authors:** Roberto Collu, Zheng Yin, Elisa Giunti, Sarah Daley, Mei Chen, Peter Morin, Richard Killick, Stephen T. C. Wong, Weiming Xia

**Affiliations:** ^1^Geriatric Research Education and Clinical Center, Bedford VA Healthcare System, Bedford, MA, United States; ^2^Department of Pharmacology, Physiology and Biophysics, Boston University Chobanian and Avedisian School of Medicine, Boston, MA, United States; ^3^T. T. and W. F. Chao Center for BRAIN, Houston Methodist Hospital, Houston Methodist Academic Institute, Houston, TX, United States; ^4^Department of Neurology, Boston University Chobanian and Avedisian School of Medicine, Boston, MA, United States; ^5^King's College London, Maurice Wohl Clinical Neuroscience Institute, London, United Kingdom; ^6^Department of Biological Sciences, University of Massachusetts Kennedy College of Science, Lowell, MA, United States

**Keywords:** Alzheimer's disease, fasudil, PS19, P301S, tau, proteomic

## Abstract

**Introduction:**

The goal of this study is to explore the pharmacological potential of the amyloid-reducing vasodilator fasudil, a selective Ras homolog (Rho)-associated kinases (ROCK) inhibitor, in the P301S tau transgenic mouse model (Line PS19) of neurodegenerative tauopathy and Alzheimer's disease (AD).

**Methods:**

We used LC-MS/MS, ELISA and bioinformatic approaches to investigate the effect of treatment with fasudil on the brain proteomic profile in PS19 tau transgenic mice. We also explored the efficacy of fasudil in reducing tau phosphorylation, and the potential beneficial and/or toxic effects of its administration in mice.

**Results:**

Proteomic profiling of mice brains exposed to fasudil revealed the activation of the mitochondrial tricarboxylic acid (TCA) cycle and blood-brain barrier (BBB) gap junction metabolic pathways. We also observed a significant negative correlation between the brain levels of phosphorylated tau (pTau) at residue 396 and both fasudil and its metabolite hydroxyfasudil.

**Conclusions:**

Our results provide evidence on the activation of proteins and pathways related to mitochondria and BBB functions by fasudil treatment and support its further development and therapeutic potential for AD.

## 1 Introduction

Alzheimer's disease (AD) is a progressive and debilitating neurodegenerative disorder with highly complex and multifactorial etiology. The disease is defined by the cerebral histopathological presence of extracellular amyloid-β (Aβ)-containing plaques, by the formation of tau-containing neurofibrillary tangles and by brain neuroinflammation that lead to widespread neurodegenerative processes that can extend for many years before the appearance of clear clinical symptoms (e.g., impairments in memory, cognition, attention and mood) (Selkoe, 2002). In the USA, around 6 million people are affected by AD, a number that is expected to double by 2050 (2023 Alzheimer's Disease Facts and Figures, 2023).

Although several target proteins contributing to its etiology have been discovered, in the past several decades only symptomatic treatments are made available (Scheltens et al., 2021). Recently, two disease modifying drugs, aducanumab (Dhillon, 2021) and lecanemab (van Dyck et al., 2022; Hoy, 2023), were approved by US Food and Drug Administration (FDA) for patients with mild cognitive impairment (MCI) or mild AD. Hence, there is a necessity for early diagnostic of MCI or mild AD and timely pharmacological approaches in order to downturn the risk and progression of the disorder. However, there is a potential of side effect of removing vascular amyloid by all seven monoclonal antibodies that aim to reduce brain amyloid load, bapineuzumab (Gao et al., 2023), solanezumab (Holdridge et al., 2023), gantenerumab (Tolar et al., 2020), crenezumab (Ostrowitzki et al., 2022), donanemab (Sims et al., 2023) and two FDA approved aducanumab and lecanemab, as patients dosed with one of above amyloid antibodies developed amyloid-related imaging abnormalities (ARIA), such as edema and micro-hemorrhage. Among all amyloid-reducing agents, we aimed to investigate a vasodilator in clinical use as a potential AD therapeutic, the Ras homolog (Rho)-associated kinases (ROCKs) inhibitor fasudil, as it unlikely induces ARIA.

ROCKs belong to the serine/threonine kinase family and activate various signaling pathways with diverse downstream effects. Two ROCKs isoforms, ROCK1 and ROCK2, have been identified and share 92% amino acid sequence while having different tissue distribution and roles. ROCK1 is ubiquitously expressed, while ROCK2 is predominantly located in the skeletal muscles and brain and show specific involvement in different physio-pathological processes including, among the others, apoptosis, insulin signaling and actin cytoskeletal reorganization (Sharma and Roy, 2020). In the central nervous system, ROCKs are involved in the regulation of neuron damage, survival and regeneration, axon guidance, and immune and glial cells (Liu et al., 2015). Therefore, it has been shown that ROCK pharmacological inhibition or inactivation promotes several physiological processes (Chong et al., 2017).

ROCKs overactivity leads to immune response, inflammation, oxidative stress, abnormal energy metabolism, neuronal loss, gliosis, and impaired synaptic transmission (Chong et al., 2017). Therefore, the ROCKs and their pharmacological inhibition has been investigated as targets for the treatment of many neurodegenerative diseases including AD (Cai et al., 2021), Parkinson's disease (Quadir et al., 2021), multiple sclerosis (Chen et al., 2015), amyotrophic lateral sclerosis (Koch et al., 2020), and Huntington's disease (Narayanan et al., 2016; Ladduwahetty et al., 2022).

Fasudil was the first ROCK inhibitor to be clinically approved, and is used for the treatment of cerebral vasospasm in Japan and China (Ono-Saito et al., 1999; Couch et al., 2010; Zhao et al., 2015; Guo et al., 2019; Hamano et al., 2020). ROCK inhibitors, including fasudil, have been shown to attenuate the symptoms and progression of neurodegenerative diseases in many pre-clinical models and in humans.

Fasudil was found to improve cognition in animal models of stroke (Satoh et al., 1996), to protect against age-related memory impairment in rats (Huentelman et al., 2009), and to improve memory in patients with cerebrovascular dementia (Kamei et al., 1996). Fasudil was shown to exert neuroprotective effects by reducing Aβ and tau deposition, oxidative stress and neuronal apoptosis, as well as improving spatial memory and restoring cognitive function in APP/PS1 mice (Elliott et al., 2018; Guo et al., 2020; Wei et al., 2021; Yan et al., 2021). Recently, the repurposing potential of fasudil has been supported by evidence showing its effect in reverting neurodegenerative-related phenotype in triple transgenic model of AD (Killick et al., 2023).

A number of reports show the ability of ROCK inhibitors to reduce the levels of phosphorylated tau and oligomeric tau protein (Hamano et al., 2020; Saray et al., 2021), pointing to the possible preventive therapeutic potential of fasudil for the treatment of tauopathies. These findings support the importance of further investigation of ROCK inhibitors for the treatment of neurodegenerative diseases.

The present study aimed to explore the potential pharmacotherapeutic effect of the ROCK inhibitor fasudil in the PS19 transgenic tau model of neurodegenerative tauopathy. We used advanced LC-MS/MS and ELISA technologies, as well as bioinformatic approaches, to deeply explore the effect of fasudil administration on the brain tau pathology and the proteomic profile of PS19 transgenic mice.

## 2 Materials and methods

### 2.1 Transgenic mouse model

PS19 mice overexpressing the T34 isoform of tau (1N4R), encoding the P301S mutant form of human microtubule-associated protein tau (MAPT), were purchased from the Jackson Lab [B6;C3-Tg(Prnp- MAPT^*^P301S)PS19Vle/J, stock #008169] and bred with non-carrier (NC) mice on a B6C3 background. NC and PS19 littermate mice were aged to 6 months and housed 4 per cage on a 12 h light/dark cycle with *ad libitum* access to food and water. All experimental procedures were performed under a protocol approved by the Institutional Animal Care and Use Committee at the Bedford VA Healthcare System (Animal Welfare Assurance Number D16-00036).

### 2.2 Drug treatment

**Figure 1:**
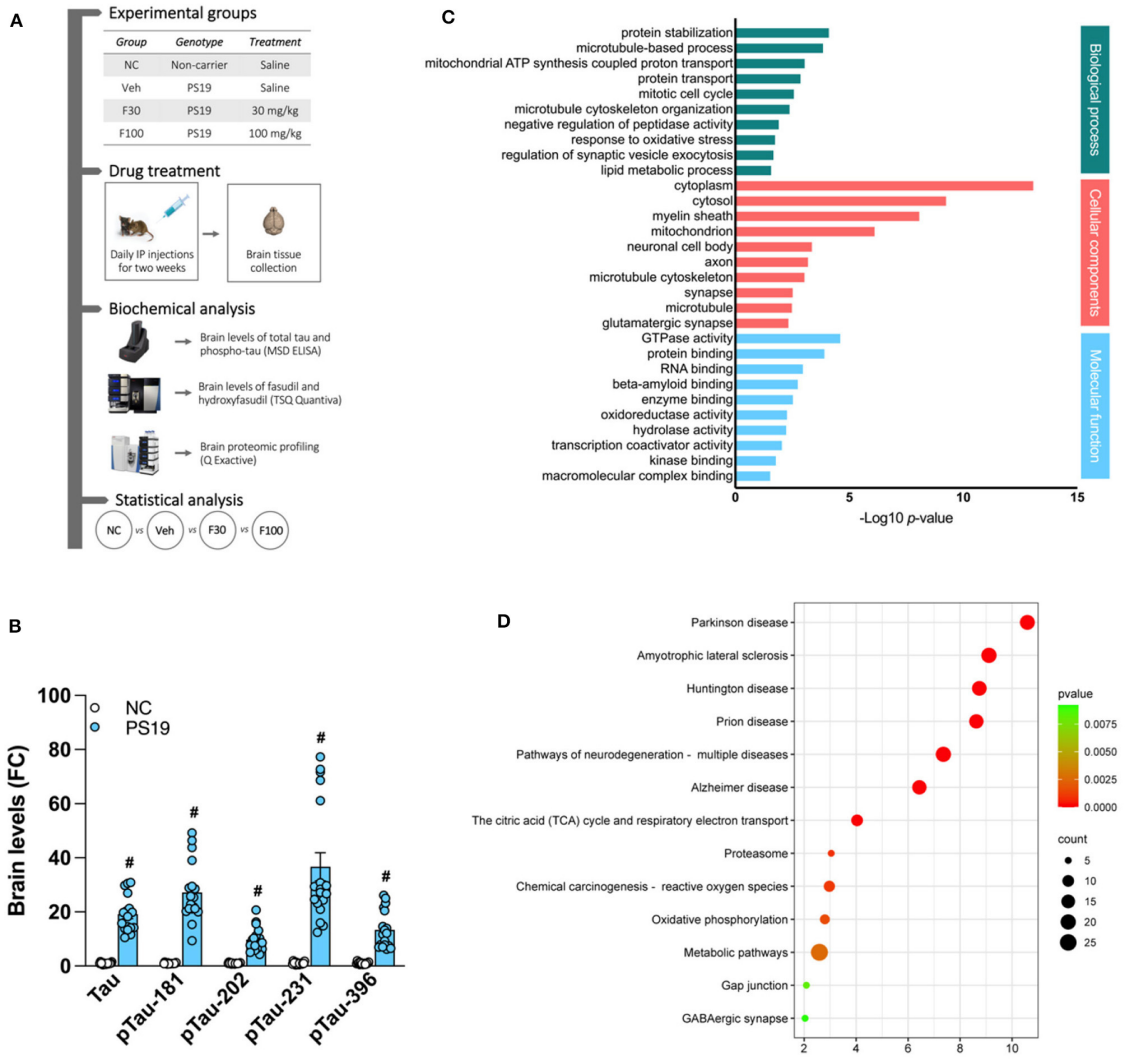
PS19 transgenic mice brain proteomic profile. **(A)** Graphical representation of the experimental timeline of this study including groups, drug treatment, biochemical and statistical analysis performed. **(B)** Levels of total tau (Tau) and phosphorylated tau (pTau)-181, −202, −231, and −396 in the brain of PS19 transgenic mice as compared to non-carrier (NC) mice measured with MSD ELISA. **(C)** Gene ontology enrichment analysis of differentially expressed proteins in the brain of PS19 as compared to NC mice. Significantly enriched biological process, cellular components, and molecular functions are represented and were obtained with DAVID. **(D)** KEGG and Reactome pathway enrichment analysis of differentially expressed proteins in the brain of PS19 as compared to NC mice. Bubble plot represents the top significant pathways.

To explore the effect of fasudil in the transgenic tau model, mice were divided into four experimental groups: (1) “NC”, vehicle treated non-carrier mice administered saline; (2) “Veh”, vehicle-treated PS19 mice administered saline; (3) “F30”, fasudil-treated PS19 mice receiving 30 mg/kg/day fasudil; (4) “F100”, fasudil-treated PS19 mice receiving 100 mg/kg/day fasudil. Saline and fasudil were administered daily by intraperitoneal (i.p.) injection for a period of 2 weeks. At the end of the treatment, animals were euthanized by exposing them to CO_2_ inhalation according to the approved protocol. The whole brain was rapidly collected, immediately frozen in liquid nitrogen and stored at −80°C until later analysis (Figure 1A). Groupings were blinded during biochemical analysis.

### 2.3 Determination of drug brain levels

Established LC-MS/MS method was adapted to determine the levels of fasudil and its active metabolite hydroxyfasudil in the brain of PS19 mice given fasudil by daily i.p. injection for a 2-week period (Collu et al., 2023). The LC-MS/MS system consists of UltiMate 3000 UHPLC automated system coupled with TSQ Quantiva triple quadrupole Mass Spectrometer (Thermo Fisher, Waltham, MA). Pre-calculated volumes of ice-cold water (ratio 1:5) were added to each brain tissue and then homogenized by TissueLyser LT (Qiagen, Valencia, CA). Tissue homogenates were centrifuged at 17,000 × g for 20 min at 4°C and the obtained supernatant was transferred into a new vial. Samples were prepared by adding 200 μl ice cold acetonitrile (ratio 1:4) containing internal standard (Ranitidine 200 ng/mL) to each sample vial containing brain lysate supernatant. Then, each sample was vortexed vigorously while keeping the sample cold by immersion into ice between the steps. Samples were then centrifuged at 12,000 x g for 10 min at 4°C, and the supernatant was aliquoted and diluted with mobile phase A, and then transferred into a HPLC vial for LC-MS/MS analysis. The chromatographic separation was performed on a Kinetex C18 column (50 x 2.1 mm, 2.6 μm particle size, Phenomenex, Torrance, CA) with mobile phase consisting of water with 0.1% formic acid (mobile phase A) and acetonitrile with 0.1% formic acid (mobile phase B), running a linear gradient from 1 to 95% for 7 min, and then maintaining at 95% for 2 min, back to 1% in 1 min, and maintaining at this proportion for 5 min to equilibrate the column. The flow rate was set to 0.30 mL/min. The M equipped with an H-ESI source was operated in the positive ionization mode with selected reaction monitoring (SRM). Ion spray voltage was 3.6 kV and ion transfer tube temperature was 325°C. The mass/charge (m/z) ratios monitored were 292/99 for fasudil, 308/99 for hydroxyfasudil and 308/99 for ranitidine. A second transition of each analyte was used for confirmation purposes.

### 2.4 Quantification of Tau and pTau brain levels

ELISA was performed to quantify brain levels of Tau and phosphorylated tau (pTau)-181, 202, 231, and 396 using our previously published method (Stathas et al., 2022). Briefly, sample lysis buffer (2% SDS, 0.5M TEAB, protease/phosphatase inhibitor cocktail; ratio 1:5 per mg of wet tissue) were added to each brain tissue and then homogenized by TissueLyser LT (Qiagen, Valencia, CA). Tissue homogenates were centrifuged at 17,000 g for 20 min at 4°C and loaded onto ELISA plates coated with the corresponding primary antibodies. Plates were read using the MSD Sector Imager 2400 (Rockville, MD, USA).

### 2.5 Analysis of brain proteomics profile

Analysis of brain proteomics profile was performed using our previously published method (Chen and Xia, 2020; Collu et al., 2023). Briefly, brain lysates were prepared using a sample lysis buffer (2% SDS, 0.5M tetraethyl-ammonium bicarbonate (TEAB), protease and phosphatase inhibitor cocktail) and the protein concentration was measured by Nanodrop One (Thermo Fisher). 100 μg of protein from each sample was reduced with tris (2-carboxyethyl) phosphine (TCEP), alkylated with iodoacetamide, precipitated with acetone and digested with trypsin overnight. Tryptic digested peptides from brain samples were labeled with TMT 10-plex reagents (Thermo Fisher) according to manufacturer's instructions. The combined TMT labeled samples were dried under SpeedVac, and then reconstituted by dilute tri-fluoroacetic acid solution followed by desalting by Oasis HLB 96-well μElution plate (Waters).

LC-MS/MS analysis was performed on a Q Exactive Orbitrap Mass Spectrometer (Thermo Fisher Scientific) coupled with a Dionex ultimate 3000 HPLC system equipped with a nano-ES ion source. The TMT labeled peptides were separated on a C18 reverse-phase capillary column (PepMap, 75 μm × 150 mm, Thermo Fisher) with linear gradients of 2–35% acetonitrile in 0.1% formic acid, at a constant flow rate of 300 nL/min for 220 min. The instrument was operated in the positive-ion mode with the ESI spray voltage set at 1.8 kV. Twenty peptide ions showing the most intense signal from each scan were selected for higher energy collision-induced dissociation (HCD)-MS/MS analysis (normalized collision energy 32) in the Orbitrap. The data were acquired using Thermo Xcalibur 3.0.63.

Raw data were processed using Proteome Discoverer (Version 2.1, Thermo Fisher Scientific). Data were searched against the mus musculus Universal Protein Resource sequence database (UniProt). The searches parameters were: trypsin digestion with two missed cleavage allowed; fixed modification, carbamidomethyl of cysteine; variable modification, oxidation of methionine, TMT 10plex (peptide labeled) for N terminus and Lys; MS tolerance, 5 ppm; MS/MS tolerance, 0.02 Da; false discovery rate (FDR) at peptide and protein levels, <0.01; and required peptide length, ≥6 amino acids. At least one unique peptide per protein group was required for identifying proteins. The relative protein abundance ratios (fold changes) between groups were calculated. The changes in protein levels were considered significant if fold change was > 1.2 (upregulated) or < 0.8 (down-regulated), and the *p* < 0.05 in two independent experiments.

### 2.6 Statistical analysis

One-way analysis of variance (ANOVA) was applied to identify significant variance in protein abundances across treatment conditions, followed by Tukey's range tests to determine the level of significance as well as directions, differences and confidence intervals for changes in protein abundances when comparing each pair of treatments. Proteins with *p* ≤ 0.05 from one-way ANOVA and *p* ≤ 0.05 for either Veh vs. NC, F30 vs. Veh or F100 vs. Veh were obtained for further analysis, creating a panel of 685 proteins. The statistical analysis was applied via statistics and machine learning toolbox in MATLAB R2021b Update 3 (9.11.01873457), and the analysis was run on a Mac Pro v2019 workstation with macOS Ventura 13.2.1. Uniprot Accession numbers for member proteins were used to query STRING database (version 11.5) and compile protein neighborhoods connected with high confidence PPIs (confidence scores ≥ 0.7). The resulted networks were visualized in Cytoscape 3.9.1, with the EnhancedGraphics plugin used to color two different halves of each node according to performances from different treatments. Statistical analysis of Tau and pTau brain levels, as well as brain concentrations of fasudil and hydroxyfasudil, was conducted using GraphPad Prism^®^ 9 (Graph Pad software, USA). Between-group comparisons were analyzed by Student's *t*-test, nonparametric Mann-Whitney U, or one-way analysis of variance (ANOVA) followed by Bonferroni correction for multiple comparisons. The Gene Ontology of identified proteins were elucidated by DAVID Bioinformatics Resources 6.8.

## 3 Results

### 3.1 PS19 transgenic mice brain tau pathology

We characterized our PS19 transgenic mouse model of AD by measuring brain levels of total tau (Tau) and pTau-181,−202, −231 and −396 and compared them with the group of NC animals. As expected, we found that, when compared to NC, the brain levels of Tau and all the pTau isoforms quantified by ELISA are significantly higher in PS19 mice (# *p* < 0.0001 vs. NC) (Figure 1B).

### 3.2 Gene ontology analysis of PS19 mice brain proteome

We performed proteomic profiling of brains from PS19 transgenic mice, without treatment with fasudil, and compared it with NC mice. We identified differentially expressed proteins and run gene ontology enrichment analysis to identify the processes significantly affected by these proteins (Figure 1C). In the category of biological process, the significantly enriched process in the brain of PS19 mice were: protein stabilization (GO:0050821; 6.99E-05), microtubule-based process (GO:0007017; 1.26E-04), mitochondrial ATP synthesis coupled proton transport (GO:0042776; 8.08E-04), protein transport (GO:0015031; 1.23E-03), mitotic cell cycle (GO:0000278; 2.41E-03), microtubule cytoskeleton organization (GO:0000226; 3.73E-03), negative regulation of peptidase activity (GO:0010466; 1.10E-02), response to oxidative stress (GO:0006979; 1.62E-02), regulation of synaptic vesicle exocytosis (GO:200030; 1.89E-02), lipid metabolic process (GO:0006629; 2.39E-02).

The analysis of cellular components revealed that the differentially expressed proteins in PS19 mice were mostly enriched in: cytoplasm (GO:0005737; 7.61E-14), cytosol (GO:0005829; 5.06E-10), myelin sheath (GO:0043209; 7.53E-09), mitochondrion (GO:0005739; 6.98E-07), neuronal cell body (GO:0043025; 3.89E-04), axon (GO:0030424; 5.85E-04), microtubule cytoskeleton (GO:0015630; 8.21E-04), synapse (GO:0045202; 2.73E-03), microtubule (GO:0005874; 2.92E-03), glutamatergic synapse (GO:0098978; 4.20E-03).

The analysis of molecular functions in PS19 mice revealed the enrichment for: GTPase activity (GO:0003924; 2.21E-05), protein binding (GO:0005515; 1.10E-04), RNA binding (GO:0003723; 9.68E-04), beta-amyloid binding (GO:0001540; 1.62E-03), enzyme binding (GO:0019899; 2.69E-03), oxidoreductase activity (GO:0016491; 4.78E-03), hydrolase activity (GO:0016787; 5.18E-03), transcription coactivator activity (GO:0003713; 8.03E-03), kinase binding (GO:0019900; 1.48E-02), macromolecular complex binding (GO:0044877; 2.62E-02) (Figure 1C).

### 3.3 Pathway analysis of PS19 mice brain proteome

In order to identify specific pathways affected by differentially expressed proteins in the brain of PS19 mice overexpressing tau we performed pathway enrichment analysis. Our analysis identified the following top significantly enriched pathways: Parkinson's disease (mmu05012; 2.57E-11), Amyotrophic lateral sclerosis (mmu05014; 7.74E-10), Huntington disease (mmu05016; 1.81E-09), Prion disease (mmu05020; 2.37E-09), Pathways of neurodegeneration - multiple diseases (mmu05022; 4.37E-08), Alzheimer's disease (mmu05010; 3.68E-07), The citric acid (TCA) cycle and respiratory electron transport (mmu1428517; 9.15E-05), Proteasome (mmu03050; 9.03E-04), Chemical carcinogenesis - reactive oxygen species (mmu05208; 1.07E-03), Oxidative phosphorylation (mmu00190; 1.59E-03), Metabolic pathways (mmu01100; 2.58E-03), Gap junction (mmu04540; 8.18E-03), GABAergic synapse (mmu04727; 9.21E-03) (Figure 1D).

### 3.4 Global panel of proteins network responding to fasudil treatment

**Figure 2:**
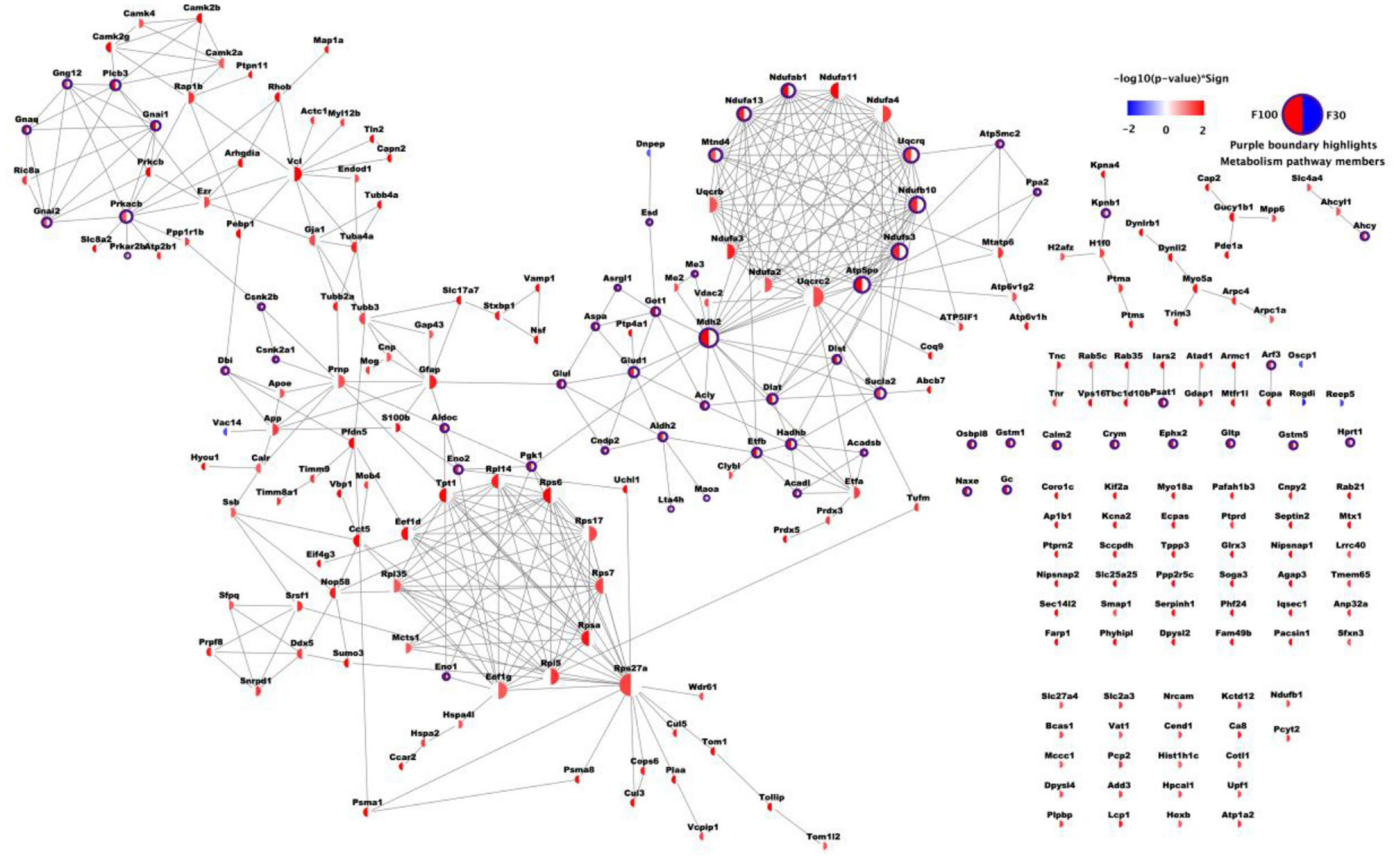
Network of differentially expressed proteins upon fasudil treatment. A total of 252 proteins are shown, with 182/253 proteins in F100 group with *p* > 0.1 for F30 vs. Veh and 70/149 proteins from F30 group with *p* > 0.1 for F100 vs. Veh. Each node represents one protein, and each node face was divided into two halves. The sector on the left was colored based on -log10 transformation of *p*-value from Tukey's test for F100 vs. Veh, while the sector on the right was colored according to F30 vs. Veh. *P*-values corresponding to up-regulation after treatment were red, while blue represented down-regulation after treatment (p ≥ 0.05 was represented in white). Each edge represents one PPI record with combined confidence score ≥ 0.7 from STRING database.

Total abundance profiles for 1,470 proteins were obtained with no missing values in any animal. Analysis of significant variance in protein abundances across three treatment conditions (i.e., Veh, F30, F100) revealed a panel of 685 proteins that were further stratified based on patterns of significant changes upon different treatments. The panel of 685 proteins responding to fasudil treatment at one or both dosages were organized into a network contains 252 proteins with significant changes after only one treatment (182 responding to F100 and 70 responding to F30), as it combines 182/253 proteins in “F100” group with *p* > 0.1 for F30 vs. Veh and 70/149 proteins from “F30” group with *p* > 0.1 for F100 vs. Veh. 181 of 252 proteins were connected through 410 high confidence PPIs (Figure 2). To get a general overview of brain protein changes in PS19 mice dosed with fasudil and to identify the most affected processes and pathways, GO functional and pathway enrichment analysis were performed.

### 3.5 Gene ontology analysis of proteins affected by fasudil

**Figure 3:**
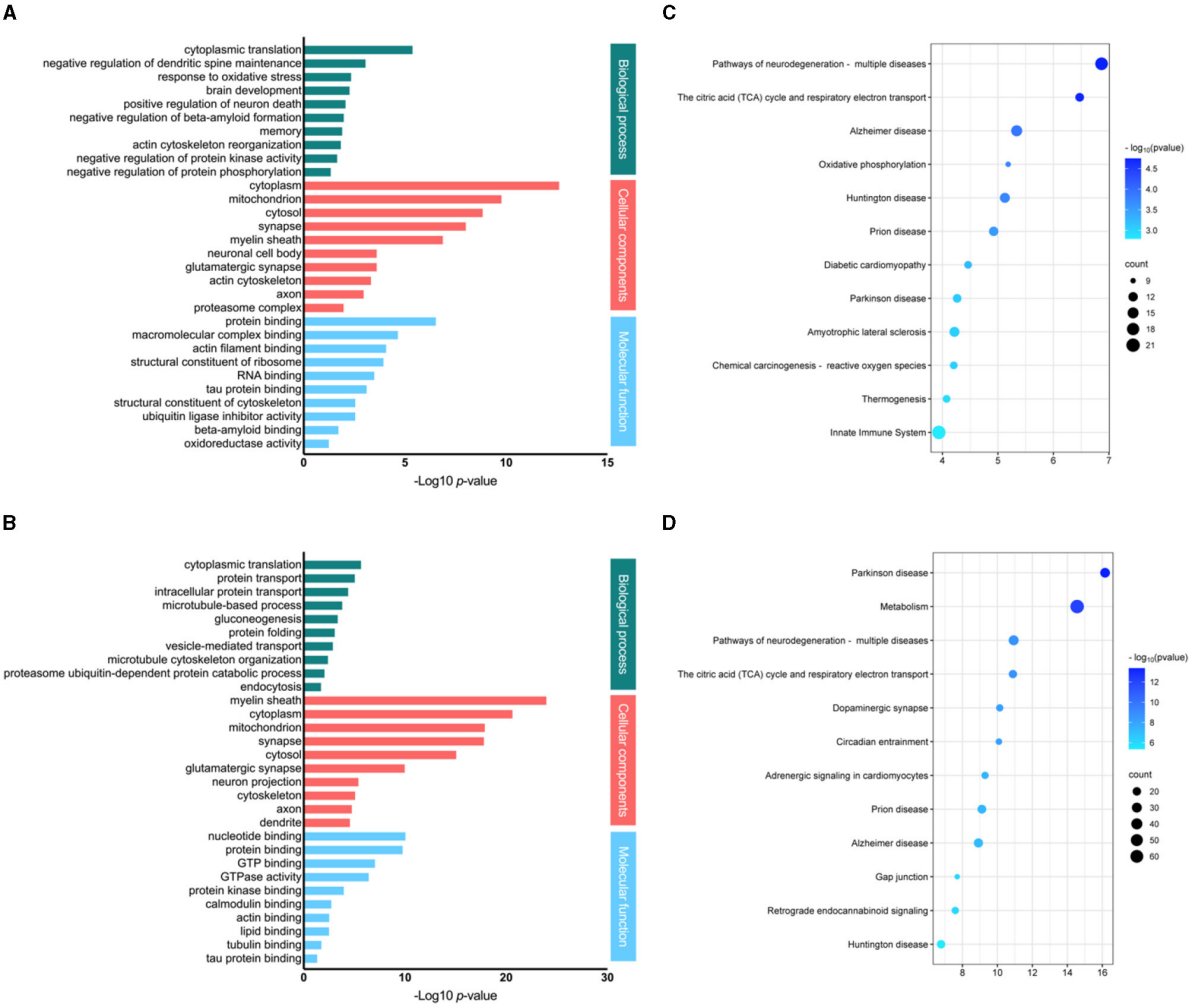
Analysis of the differentially expressed proteins responding to fasudil. Gene ontology and pathway enrichment analysis of differentially expressed proteins in the brain of PS19 transgenic mice dosed with fasudil 30 mg/kg/day [**(A, C)** respectively] or 100 mg/kg/day [**(B, D)** respectively] as compared to the control group (Veh, saline). The most significantly enriched biological process, cellular components and molecular functions are represented by bar graphs, while the top significantly affected pathways are represented by bubble plots.

All differentially regulated brain proteins (including up- and down-regulated proteins) in response to low (30 mg/kg) and high (100 mg/kg) dosage of fasudil were analyzed with DAVID. The identified proteins, were classified into different groups according to biological process, cellular components and molecular function (Figure 3). In the category of biological process, the significantly enriched process in response to the low dose of fasudil (30 mg/kg; Figure 3A) were: cytoplasmatic translation (3.7E-06), negative regulation of dendritic spine maintenance (7.8E-04), response to oxidative stress (4.0E-03), brain development (4.8E-03), positive regulation of neuron death (7.6E-03), negative regulation of beta-amyloid formation (9.2E-03), memory (1.1E-02), actin cytoskeleton reorganization (1.3E-02), negative regulation of protein kinase activity (2.0E-02), negative regulation of protein phosphorylation (4.1E-02). As a response to the high dose of fasudil (100 mg/kg; Figure 3B), the significantly enriched biological process were: cytoplasmatic translation (1.6E-06), protein transport (7.1E-06), intracellular protein transport (3.2E-05), microtubule-based process (1.2E-04), gluconeogenesis (3.4E-04), protein folding (6.8E-04), vesicle-mediated transport (1.0E-03), microtubule cytoskeleton organization (3.2E-03), proteasome ubiquitin-dependent protein catabolic process (7.0E-03), endocytosis (1.5E-02).

The cellular component analysis revealed that the brain proteins responding to the low dose of fasudil (Figure 3A) were enriched in the cytoplasm (2.1E-13), mitochondrion (1.5E-10), cytosol (1.3E-09), synapse (8.7E-09), myelin sheath (1.2E-07), neuronal cell body (2.2E-04), glutamatergic synapse (2.2E-04), actin cytoskeleton (4.2E-04), axon (9.7E-04) and proteasome complex (9.5E-03); while, those responding to the high dose of fasudil (Figure 3B) were enriched in the myelin sheath (8.3E-25), cytoplasm (1.9E-21), mitochondrion (1.0E-18), synapse (1.2E-18), cytosol (6.8E-16), glutamatergic synapse (8.3E-11), neuron projection (3.0E-06), cytoskeleton (6.5E-06), axon (1.4E-05) and dendrite (2.2E-05).

The analysis of molecular functions enriched by the treatment with fasudil showed, as a result of the low dose (Figure 3A), protein binding (2.6E-07), macromolecular complex binding (2.0E-05), actin filament binding (7.6E-05), structural constituent of ribosome (1.0E-04), RNA binding (2.9E-04), tau protein binding (7.0E-04), structural constituent of cytoskeleton (2.5E-03), ubiquitin ligase inhibitor activity (2.5E-03), beta-amyloid binding (1.7E-02), oxidoreductase activity (4.8E-02). Whereas, as a result of the high dose (Figure 3B), we observed nucleotide binding (7.0E-11), protein binding (1.3E-10), GTP binding (7.2E-08), GTPase activity (3.0E-07), protein kinase binding (8.5E-05), calmodulin binding (1.5E-03), acting binding (2.3E-03), lipid binding (2.5E-03), tubulin binding (1.4E-02), tau protein binding (3.7E-02).

### 3.6 Pathways modulated by fasudil treatment

The differentially expressed proteins identified in the brain of PS19 mice in response to fasudil treatment were analyzed to identify overrepresented signaling pathways. Top significantly enriched pathways responding to fasudil treatment are represented (Figure 3). The top enriched pathways in the F30 group were: Pathways of neurodegeneration—multiple diseases (q-value: 1.8E-05), The citric acid (TCA) cycle and respiratory electron transport (q-value: 2.0E-05), Alzheimer's disease (q-value: 1.3E-04), Oxidative phosphorylation (q-value: 1.8E-04), Huntington disease (q-value: 1.9E-04), Prion disease (q-value: 2.9E-04), Diabetic cardiomyopathy (q-value: 5.8E-04), Parkinson's disease (q-value: 8.5E-04), Amyotrophic lateral sclerosis (q-value: 9.1E-04), Chemical carcinogenesis—reactive oxygen species (q-value: 9.1E-04), Thermogenesis (q-value: 1.2E-03), Innate Immune System (q-value: 1.6E-03) (Figure 3C); while in the F100 group were: Parkinson's disease (q-value: 4.3E-14), Metabolism (q-value: 8.5E-13), Pathways of neurodegeneration—multiple disease (q-value: 2.0E-09), The citric acid (TCA) cycle and respiratory electron transport (q-value: 2.0E-09), Dopaminergic synapse (q-value: 7.3E-09), Circadian entrainment (q-value: 7.3E-09), Adrenergic signaling in cardiomyocytes (q-value: 3.9E-08), Prion disease (q-value: 5.3E-08), Alzheimer's disease (q-value: 7.5E-08), Gap junction (q-value: 7.7E-07), Retrograde endocannabinoid signaling (q-value: 9.4E-07), Huntington disease (q-value: 4.3E-06) (Figure 3D).

### 3.7 Fasudil affects mitochondrial proteins expression

**Figure 4:**
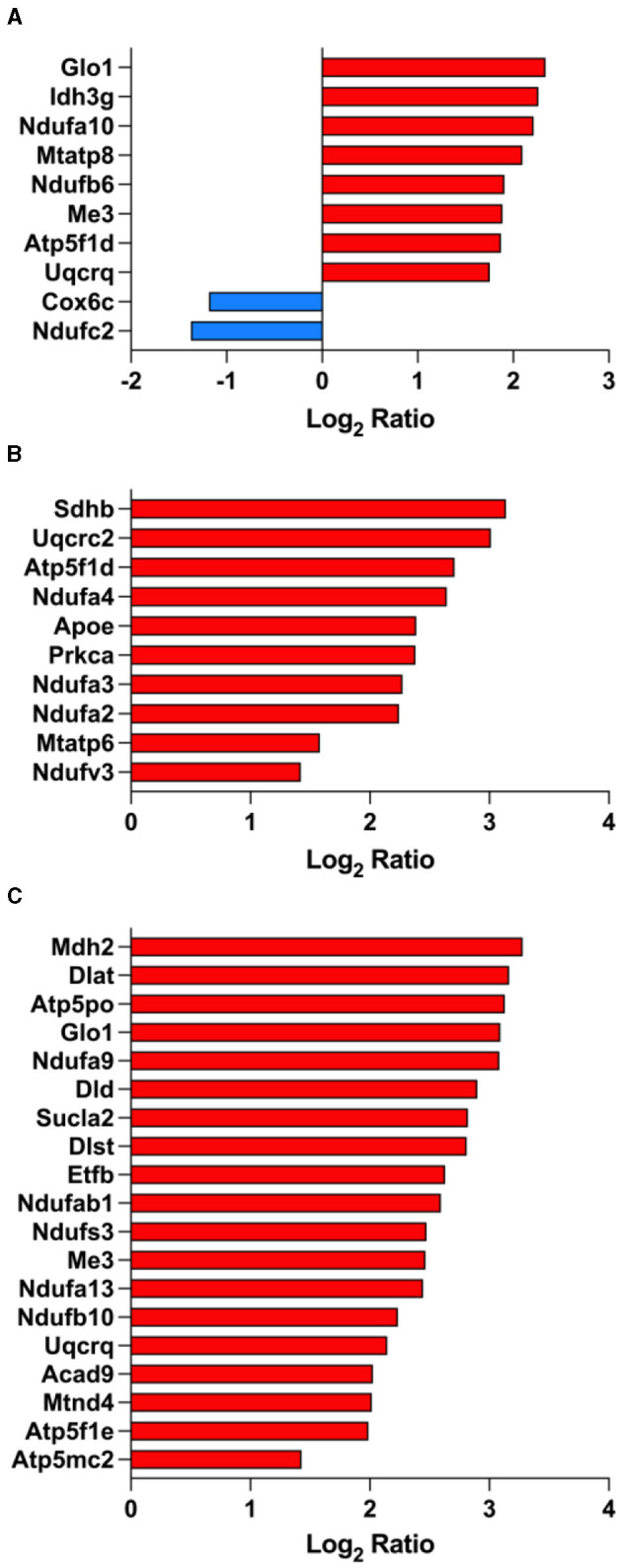
Fasudil affects mitochondrial proteins expression. Proteins showing a significant differential expression in the brain of PS19 mice compared to NC mice **(A)**, PS19 mice dosed with 30 mg/kg/day fasudil compared to vehicle-dosed mice **(B)**, and PS19 mice dosed with 100 mg/kg/day fasudil compared to vehicle-dosed mice **(C)**. The significant proteins were obtained from the Reactome pathway for TCA cycle (R-MMU-148517, the citric acid cycle and respiratory electron transport) ranked among the top significantly enriched pathways in PS19 transgenic mice and after treatment with fasudil.

As shown above, the citric acid (TCA) cycle and respiratory electron transport pathway ranked among the top significantly enriched pathways in PS19 mice and as a result of treatment with fasudil and several mitochondrial proteins were differentially expressed (Figure 4). When comparing PS19 mice with NC mice we found eight proteins that were significantly up-regulated: Glo1 (Lactoylglutathione lyase; Q9CPU0), Idh3g (Isocitrate dehydrogenase [NAD] subunit gamma 1; P70404), Ndufa10 (NADH dehydrogenase [ubiquinone] 1 alpha subcomplex subunit 10; Q99LC3), Mtatp8 (ATP synthase protein 8; P03930), Ndufb6 (NADH dehydrogenase [ubiquinone] 1 beta subcomplex subunit 6; Q3UIU2), Me3 (NADP-dependent malic enzyme; Q8BMF3), Atp5f1d (ATP synthase subunit delta; Q9D3D9), Uqcrq (Cytochrome b-c1 complex subunit 8; Q9CQ69); while 2 proteins were significantly down-regulated in PS19 mice: Cox6c (Cytochrome c oxidase subunit 6C; Q9CPQ1), Ndufc2 (NADH dehydrogenase [ubiquinone] 1 subunit C2; Q9CQ54) (Figure 4A).

We explored the effect of fasudil and identified 10 up-regulated mitochondrial proteins whose expression was affected by the lower dose (30 mg/kg/day): Sdhb (Succinate dehydrogenase [ubiquinone] iron-sulfur subunit; Q9CQA3), Uqcrc2 (Cytochrome b-c1 complex subunit 2; Q9DB77), Atp5f1d (ATP synthase subunit delta; Q9D3D9), Ndufa4 (Cytochrome c oxidase subunit NDUFA4; Q62425), Prkca (Protein kinase C alpha type; P20444), ApoE (Apolipoprotein E; P08226), Ndufa3 (NADH dehydrogenase [ubiquinone] 1 alpha subcomplex subunit 3; Q9CQ91), Ndufa2 (NADH dehydrogenase [ubiquinone] 1 alpha subcomplex subunit 2; Q9CQ75), Mtatp6 (ATP synthase subunit a; P00848), Ndufv3 (NADH dehydrogenase [ubiquinone] flavoprotein 3; Q8BK30) (Figure 4B).

Similarly, we identified 19 up-regulated mitochondrial proteins after treatment with the higher dose of fasudil (100 mg/kg/day): Mdh2 (Malate dehydrogenase; P08249), Dlat (Dihydrolipoyllysine-residue acetyltransferase component of pyruvate dehydrogenase complex; Q8BMF4), Atp5po (ATP synthase subunit O; Q9DB20), Glo1 (Lactoylglutathione lyase; Q9CPU0), Ndufa9 (NADH dehydrogenase [ubiquinone] 1 alpha subcomplex subunit 9; Q9DC69), Dld (Dihydrolipoyl dehydrogenase; O08749), Sucla2 (Succinate–CoA ligase [ADP-forming] subunit beta; Q9Z2I9), Dlst (Dihydrolipoyllysine-residue succinyltransferase component of 2-oxoglutarate dehydrogenase complex; Q9D2G2), Etfb (Electron transfer flavoprotein subunit beta; Q9DCW4), Ndufab1 (Acyl carrier protein; Q9CR21), Ndufs3 (NADH dehydrogenase [ubiquinone] iron-sulfur protein 3; Q9DCT2), Me3 (NADP-dependent malic enzyme; Q8BMF3), Ndufa13 (NADH dehydrogenase [ubiquinone] 1 alpha subcomplex subunit 13; Q9ERS2), Ndufb10 (NADH dehydrogenase [ubiquinone] 1 beta subcomplex subunit 10; Q9DCS9), Uqcrq (Cytochrome b-c1 complex subunit 8; Q9CQ69), Acad9 (Acyl-CoA dehydrogenase family member 9; Q8JZN5), Mtnd4 (NADH-ubiquinone oxidoreductase chain 4; P03911), Atp5f1e (ATP synthase subunit epsilon, mitochondrial; P56382), Atp5mc2 (ATP synthase F(0) complex subunit C2; P56383) (Figure 4C).

### 3.8 Fasudil affects gup junction proteins expression

**Figure 5:**
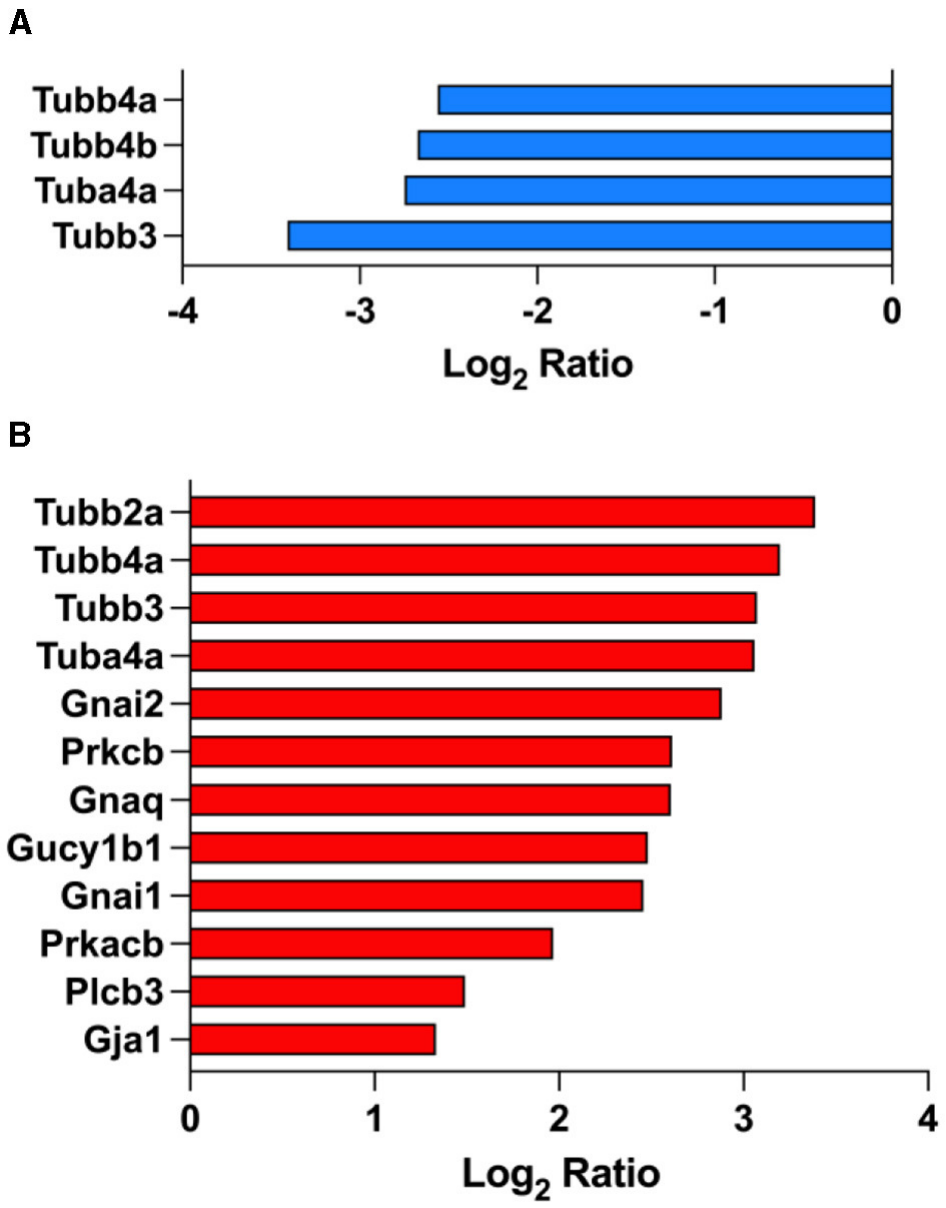
Gap junction proteins are affected by fasudil treatment. Differentially expressed proteins in the brain of PS19 mice compared to NC mice **(A)**, and PS19 mice dosed with 100 mg/kg/day fasudil compared to vehicle-dosed mice **(B)** obtained from the KEGG pathway for Gap junction (MMU04540) ranked among the top significantly enriched pathways in PS19 transgenic mice and after treatment with fasudil.

Our data revealed that gup junction proteins were significantly affected by the overexpression of tau in the brain on PS19 mice (Figure 5). We identified 4 down-regulated gup junction proteins in PS19 animals when compared to NC animals: Tubb4a (Tubulin beta-4A chain; Q9D6F9), Tubb4b (Tubulin beta-4B chain; P68372), Tuba4a (Tubulin alpha-4A chain; P68368), Tubb3 (Tubulin beta-3 chain; Q9ERD7) (Figure 5A). Treatment with fasudil induced the up-regulation of 12 gup junction proteins in the brain of PS19 mice dosed with the higher dose (100 mg/kg/day): Tubb2a (Tubulin beta-2A chain; Q7TMM9), Tubb4a (Tubulin beta-4A chain; Q9D6F9), Tubb3 (Tubulin beta-3 chain; Q9ERD7), Tuba4a (Tubulin alpha-4A chain; P68368), Gnai2 (Guanine nucleotide-binding protein G(i) subunit alpha-2; P08752), Prkcb (Protein kinase C beta type; P68404), Gnaq (Guanine nucleotide-binding protein G(q) subunit alpha; P21279), Gucy1b1 (Guanylate cyclase soluble subunit beta-1; O54865), Gnai1 (Guanine nucleotide-binding protein G(i) subunit alpha-1; B2RSH2), Prkacb (cAMP-dependent protein kinase catalytic subunit beta; P68181), Plcb3 (1-phosphatidylinositol 4,5-bisphosphate phosphodiesterase beta-3; P51432), Gja1 (Gap junction alpha-1 protein; P23242) (Figure 5B).

### 3.9 Effect of fasudil on the brain tau pathology

**Figure 6:**
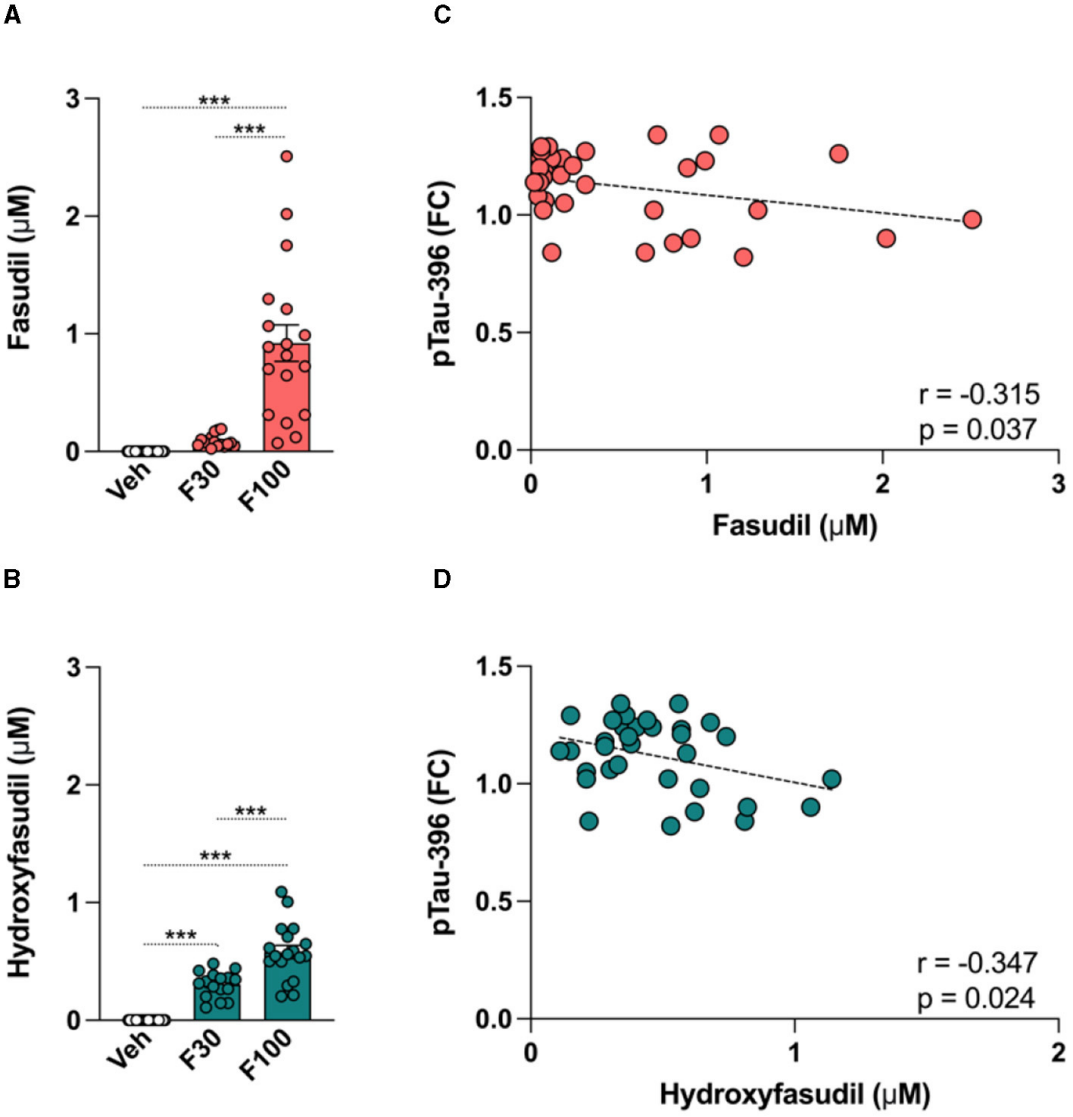
Effect of fasudil on the brain tau pathology in PS19 mice. PS19 transgenic mice received daily intraperitoneal injections with vehicle (Veh), fasudil 30 mg/kg (F30), and fasudil 100 mg/kg (F100) for 2 weeks. At the end of the treatment whole brains were collected and processed to quantify levels of fasudil and its metabolite hydroxyfasudil, as well as brain concentrations of total tau (Tau) and phosphorylated tau (pTau) isoforms. Brain levels of fasudil **(A)** and hydroxyfasudil **(B)** increased in a dose dependent way in PS19 mice administered 30 and 100 mg/kg/day. Brain levels of pTau-396 showed a significant negative correlation with fasudil **(C)** and hydroxyfasudil **(D)**. Data are presented as mean ± standard error of means (SEM; ***p < 0.001).

The brain concentration of fasudil and its metabolite hydroxyfasudil were determined by LC-MS/MS analysis in PS19 mice given fasudil at 30 or 100 mg/kg daily for a 2-week period (Figure 6). The pharmacological treatment significantly and dose dependently affected the levels of fasudil [One-way ANOVA: F_(2,50)_ = 31.14, *p* < 0.0001] and hydroxyfasudil [One-way ANOVA: F_(2,50)_ = 66.94, *p* < 0.0001] in the brain of PS19 mice. The levels of fasudil and hydroxyfasudil were significantly higher in the brain of PS19 mice administered fasudil 100 mg/kg/day (F100) as compared to the group of mice administrated 30 mg/kg/day (F30; *p* < 0.0001; Figures 6A, B).

To explore the effect of fasudil on the tau pathology in PS19 transgenic mice, ELISA analysis was performed and brain levels of Tau and pTau-181, 202, and 396 were quantified after fasudil administration. When values from each group of animals were averaged, no significant change in the levels of Tau and pTau was observed after treatment. We searched for potential correlations between the brain concentration of fasudil and hydroxyfasudil with the brain levels of Tau and different pTau isoforms. Our analysis revealed that the levels of pTau-396 were negatively correlated with the brain levels of fasudil (*r* = −0.315, *p* = 0.037; Figure 6C) and hydroxyfasudil (*r* = −0.347, *p* = 0.024; Figure 6D) in PS19 mice dosed with fasudil.

## 4 Discussion

In this study we explored the effect of the ROCK inhibitor fasudil on the brain proteomic profile and tau pathology in PS19 tau transgenic mice modeling tauopathies and AD.

As expected, at the age of 6 months we found marked increase of total tau brain levels in our PS19 transgenic mice as compared to non-transgenic littermates. Increased accumulation of hyperphosphorylated tau in the brain of PS19 mice was also significantly evident at the same age. As well known, tau is a microtubule-associated protein that, under physiological conditions, stabilizes neuronal microtubules and axonal transport. However, in pathological conditions, abnormal tau accumulation and aggregation leads to neurodegeneration (Castellani, 2020). Accordingly, when comparing differentially regulated proteins in the brain, we found significant proteomic changes related to microtubule-based process in PS19 transgenic mice.

As compared to non-transgenic animals, our analysis revealed that the tau protein in the brain of PS19 mice was significantly hyperphosphorylated at different serine/threonine sites including −181, −202, −231 and −396, that are highly associated with different tauopathies (Buée et al., 2000). Interestingly, we found enrichment for several pathways related to different neurodegenerative diseases such as Parkinson's disease, Amyotrophic lateral sclerosis, Huntington's disease, Prion's disease, and AD, indicating that the tau hyperphosphorylation observed in the brain of PS19 transgenic mice reflects the status of different tauopathies, thus validating the use of this model to study different neurodegenerative diseases.

In agreement with previous evidence, our investigation identified various levels of enrichment for different mitochondrial energy production pathways and sub-systems in PS19 mice (Tsumagari et al., 2022). In particular, we found the citric acid (TCA) cycle and respiratory electron transport pathway, as well as the oxidative phosphorylation pathway among the top significantly affected pathways in the brain of PS19 mice. Molecular changes inducing altered mitochondrial function, oxidative stress damage, neuronal death, neuroinflammation, as well as modifications in brain lipids composition have been observed in advanced stages of tau pathology disease in PS19 transgenic mice (López-González et al., 2015; Tsumagari et al., 2022). Indeed, behavioral abnormalities together with impaired expression and activity of mitochondrial enzymes specifically involved in the formation of reactive oxygen species were detected in PS19 mice at 7 months of age (Dumont et al., 2011). Accordingly, we found that differentially regulated proteins in the brain of our 6 months old PS19 mice were markedly linked to mitochondrial components and functions, response to oxidative stress, and brain lipid metabolic processes, confirming the development of these brain impairments in aged transgenic tau mice.

The mitochondrial complexes have been studied as therapeutic targets for multiple human diseases including AD (Trushina et al., 2022). Quantitative proteomic profiling of AD brains identified differentially altered mitochondrial complexes proteins as potential drivers of AD neuropathology (Adav et al., 2019). Also, a mitochondrial signature in AD has been proposed by the identification of key differentially expressed proteins and candidate biomarkers indicative of mitochondrial dysfunction in the cortex, cerebrospinal fluid and serum from AD patients (Wang et al., 2020). Indeed, different studies have demonstrated mitochondrial dysfunctions and impaired activity of related metabolic pathways such as the TCA cycle in AD, suggesting that therapies improving mitochondria functions might have a future clinical efficacy for AD patients (Atamna and Frey, 2007; Chhimpa et al., 2023).

Interestingly, evidence show that improving the impaired mitochondrial morphology and function in the brain of 11 months old PS19 mice exerted several neuroprotective effects by reducing toxic tau accumulation, attenuating neuronal loss and synaptic degeneration, as well as neuroinflammation, and improving cognitive function in the Barnes maze test (Wang et al., 2021). Pharmacological treatment with a radix extract has been shown to reduce AD markers in models *in vitro* and *in vivo* trough the activation of genes in the TCA cycle and oxidative phosphorylation pathways (Jo et al., 2022).

In our PS19 model we found that fasudil, at both doses tested, significantly affected the regulation of several proteins in the TCA cycle and oxidative phosphorylation pathways. Integrative genomic analysis identified mitochondrial dysfunction underlying AD onset mediated by oxidative phosphorylation and retrograde endocannabinoid signaling pathways (Chen et al., 2022). Interestingly, the up-regulation of proteins involved in the above-mentioned pathways (i.e., Ndufab1, Sdhb, Uqcrc2) in our PS19 mice exposed to fasudil treatment highlight its promising potential by targeting these molecular signatures of AD pathogenesis. In particular, in these AD brains Ndufa4 and Ndufa9 were altered, and both proteins expression was significantly impacted by fasudil treatment in our PS19 mice, suggesting the effect of fasudil on the impaired mitochondrial function. ROCK inhibition by fasudil has shown protective effects in a 3-Nitropropionic acid (3-NP)-induced neurotoxicity model of neurodegeneration and significantly ameliorated neurological and motor abnormalities, while improving neuronal apoptosis and severe mitochondrial dysfunctions (Ahmed et al., 2016).

Central and peripheral alterations of TCA cycle components has been demonstrated in brains and blood from AD patients (Jia et al., 2023). Interestingly, some of the TCA cycle markers that were down-regulated in different AD brain areas, such as Mdh2, Sdhb, Sucla2, and Dld, were significantly up-regulated in the brain of our PS19 transgenic mice receiving fasudil treatment. Cox6c plays important role in oxidative phosphorylation and energy production and a decrease of its expression and activity has been observed in the brain of AD patients and associated with impaired metabolic activity and neuronal loss (Wang et al., 2022). Similarly, we also observed a decrease expression of Cox6c in PS19 mice, that was reverted to control animals by the treatment with fasudil.

We also found the gap junction pathway among the top significantly enriched pathways in PS19 transgenic mice and identified several of its proteins that were differentially regulated after treatment with fasudil. Evidence suggest that gap junctions are involved in the regulation of blood-brain barrier (BBB) glial cells intercellular signaling process and are responsible for the communication between neurons and glia gaining crucial roles in neuroinflammation, cell death and intracerebral hemorrhage processes (Zhang et al., 2021). In particular, Spéder and Brand (2014) showed that gap junction proteins are required in the BBB glia to coordinate nutrient-dependent calcium oscillations and for the secretion of insulin-like peptides.

Evidence show that fasudil has a protective effect on the integrity and permeability of the BBB by acting directly on endothelial cells and has been proposed as a novel therapy for multiple sclerosis (Huang et al., 2011; Sato et al., 2022). In our tau model, fasudil was able to revert the significant down-regulation of gap junctions proteins observed in PS19 mice, as proteins such as Tubb4a, Tuba4a, and Tubb3 were significantly up-regulated after treatment. Among the other proteins affected by fasudil administration, Prkcb was found to be involved in the hyperglycemia-induced BBB dysfunction in an *in vitro* human model of brain microvasculature and BBB (Shao and Bayraktutan, 2013). The down-regulation of Gja1 has been observed in the brain of mice showing impaired BBB permeability after being exposed to a hypergravity-induced model, thus suggesting a link with the dysregulation of tight junctions of BBB endothelial cells (Dubayle et al., 2023). The effect of fasudil on gap junction proteins observed in our PS19 transgenic tau mice supports its involvement in BBB-related neuropathological processes and its potential use as pharmacological approach for AD and other tauopathies.

The effect of fasudil on memory and cognitive impairments has been previously tested in different pre-clinical models. Chronic administration of fasudil to adult OPHN1 mice modeling intellectual disability was able to revert recognition memory deficits, reduce ventricle enlargement and partially restore working and spatial memory (Meziane et al., 2016). Moreover, fasudil treatment improved cognitive function in the object-in-place memory task and the Morris Water Maze test following status convulsion in rats (He et al., 2017). In 8 months old APP/PS1 mice modeling AD fasudil restored cognitive function by improving learning and memory, but also increasing antioxidative response and reducing apoptosis in the hippocampus (Wei et al., 2021; Yan et al., 2021).

In the PS19 model, memory impairments start early in life and are associated with significant reduction of dendritic spines (Xu et al., 2014). Although we did not perform behavioral tests to assess the effect of fasudil on cognitive functions, our proteomic analysis showed the significant effect of ROCK inhibition on memory processes. We found proteins involved in memory and synapses structure and functions that were significantly enriched after treatment with fasudil. In this regard, protective properties of ROCK inhibition by fasudil on synaptic structure and function, and learning-memory abilities have been demonstrated (Hou et al., 2012; Saal et al., 2021). Together with the above-mentioned evidence, our omics results further support the potentials of the ROCK inhibition in improving memory and cognitive-related processes in tauopathies that deserves further investigation.

We recently found that fasudil was able to reduce the increase of pTau at different residues (i.e., pTau-202, −231, and −396) in Alzheimer's induced pluripotent stem cell-derived neurospheroids via clusterin (clu) and the AKT serine/threonine-protein kinase 1 (AKT1) (Giunti et al., 2023). In our PS19 mice, treatment with fasudil significantly affected tau phosphorylation in the brain in a dose dependent manner. When averaged levels of Tau and pTau were calculated from each treatment group, minor differences were observed, while when individual animals were analyzed for their drug exposure and efficacy, we found a statistically significant effect of fasudil on pTau-396. In particular, we found that the negative correlation between brain levels of pTau-396 and the brain levels of both fasudil and its metabolite hydroxyfasudil reached statistical significance. The observed effect of fasudil on pTau might reflect the impact of the drug on existing brain tau pathology as we administered fasudil to PS19 mice at 6 months of age with already established brain tau accumulation (Yoshiyama et al., 2007).

Results from different clinical trials showed the efficacy and safety of ROCK inhibitors use for different conditions. Among them, a randomized clinical trial on fasudil hydrochloride has shown significant therapeutic effects for the treatment of cerebral vasospasm following subarachnoid hemorrhage (Zhao et al., 2006). Another prospective study demonstrated the efficacy of fasudil administration in improving mortality of patients with pulmonary hypertension and acute right heart failure (Jiang et al., 2015). In both studies, fasudil was administered in patients at 30 mg three times daily, and no severe adverse effects or reactions were observed. Fasudil has been also proposed as a promising therapy for amyotrophic lateral sclerosis and a phase IIa clinical trial to evaluate its efficacy in early stages of the disease is currently ongoing (Lingor et al., 2019; Koch et al., 2020).

In our pre-clinical study, we have investigated the effect of fasudil administration at two different dosages, 30 and 100 mg/kg/day, and we've not observed any specific behavioral side effects or the activation of pathways of toxicity. However, when comparing the effect of the two doses tested we have also identified proteins showing opposite regulation (Supplementary Figure 1) that might explain potential adverse effects of the use of high doses. Among those showing similar regulation (Table 1), we have found key proteins involved in the regulation of neuronal differentiation (Ndrg3), neuronal survival (Dynlrb1), antioxidant activity important for neuron survival (Lancl1), cognitive function (Tsnax), as well as hippocampal neurons development and spatial memory (Rab35) (Huang et al., 2014, 2023; Terenzio et al., 2020; Maejima et al., 2023; Xu et al., 2023). All these proteins were up-regulated by both fasudil doses tested and might suggest consistent beneficial proteome regulations in PS19 mice.

**Table 1:** Differentially expressed proteins in PS19 mice.

**Accession**	**Protein**	**Description**	**Veh**	**F30**	**F100**
P62627	Dynlrb1	Dynein light chain roadblock-type 1	↓	↑	↑
O35127	Grcc10	Protein C10	↓	↑	↑
Q9QZE7	Tsnax	Translin-associated protein X	=	↑	↑
Q9D8W7	Ociad2	OCIA domain-containing protein 2	=	↑	↑
Q3UPH1	Prrc1	Protein PRRC1	=	↑	↑
Q9QYF9	Ndrg3	Protein NDRG3	=	↑	↑
Q9D6J5	Ndufb8	NADH dehydrogenase [ubiquinone] 1 beta subcomplex subunit 8	=	↑	↑
P84084	Arf5	ADP-ribosylation factor 5	=	↑	↑
O89112	Lancl1	Glutathione S-transferase LANCL1	=	↑	↑
P23242	Gja1	Gap junction alpha-1 protein	=	↑	↑
P62897	Cycs	Cytochrome c, somatic	=	↑	↑
P68033	Actc1	Actin, alpha cardiac muscle 1	=	↑	↑
Q6PHN9	Rab35	Ras-related protein Rab-35	=	↑	↑
Q02053	Uba1	Ubiquitin-like modifier-activating enzyme 1	=	↑	↑

List of differentially expressed proteins showing significant changes in PS19 mice as compared to NC (Veh vs. NC) and after treatment with both 30 mg/kg/day (F30 vs. Veh) and 100 mg/kg/day (F100 vs. Veh) (↑ indicates up-regulation, ratio > 1.2; ↓ indicates down-regulation, ratio < 0.8; = indicates no change).

Further studies aiming at investigating the molecular and behavioral effects of fasudil at early stages of tau-related neuropathology development, differentiating female and male subjects' responses to fasudil treatment, and focusing on specific brain regions where the tau-mediated neuropathology is more pronounced will be needed.

Overall, our data supports the protective effect of the ROCK inhibition mediated by fasudil in the PS19 tau transgenic mouse model. Furthermore, our results expand the current knowledge on the neuroprotective activity of fasudil that might be exerted by improving BBB gap junction modulation and mitochondrial functions and support its potential use for the treatment of different tauopathies and AD conditions.

## Data availability statement

The original contributions presented in the study are included in the article/Supplementary material, further inquiries can be directed to the corresponding author.

## Ethics statement

The animal study was approved by Bedford VA Healthcare System IACUC. The study was conducted in accordance with the local legislation and institutional requirements.

## Author contributions

RC: Formal analysis, Data curation, Writing – original draft, Writing – review & editing; ZY: Formal analysis, Writing – review & editing; EG: Formal analysis, Writing – review & editing; SD: Formal analysis; Writing – review and editing; MC: Formal analysis; Writing – review and editing; PM: Writing – review & editing; RK: Writing – review & editing; SW: Writing – review & editing; WX: Funding acquisition, Writing – review & editing. All authors approved the manuscript and gave their consent for submission and publication.
