# Erratum: Effect of the ROCK inhibitor fasudil on the brain proteomic profile in the tau transgenic mouse model of Alzheimer's disease

**DOI:** 10.3389/fnagi.2024.1409164

**Published:** 2024-04-10

**Authors:** Frontiers Production Office

**Affiliations:** Frontiers Media SA, Lausanne, Switzerland

**Keywords:** Alzheimer's disease, fasudil, PS19, P301S, tau, proteomic

Due to a production error, there was a mistake in Figure 1 as published. Figure 1B is supposed to be Figure 1C and Figure 1C is supposed to be Figure 1B.

The correct figure appears below:

**Figure 1:**
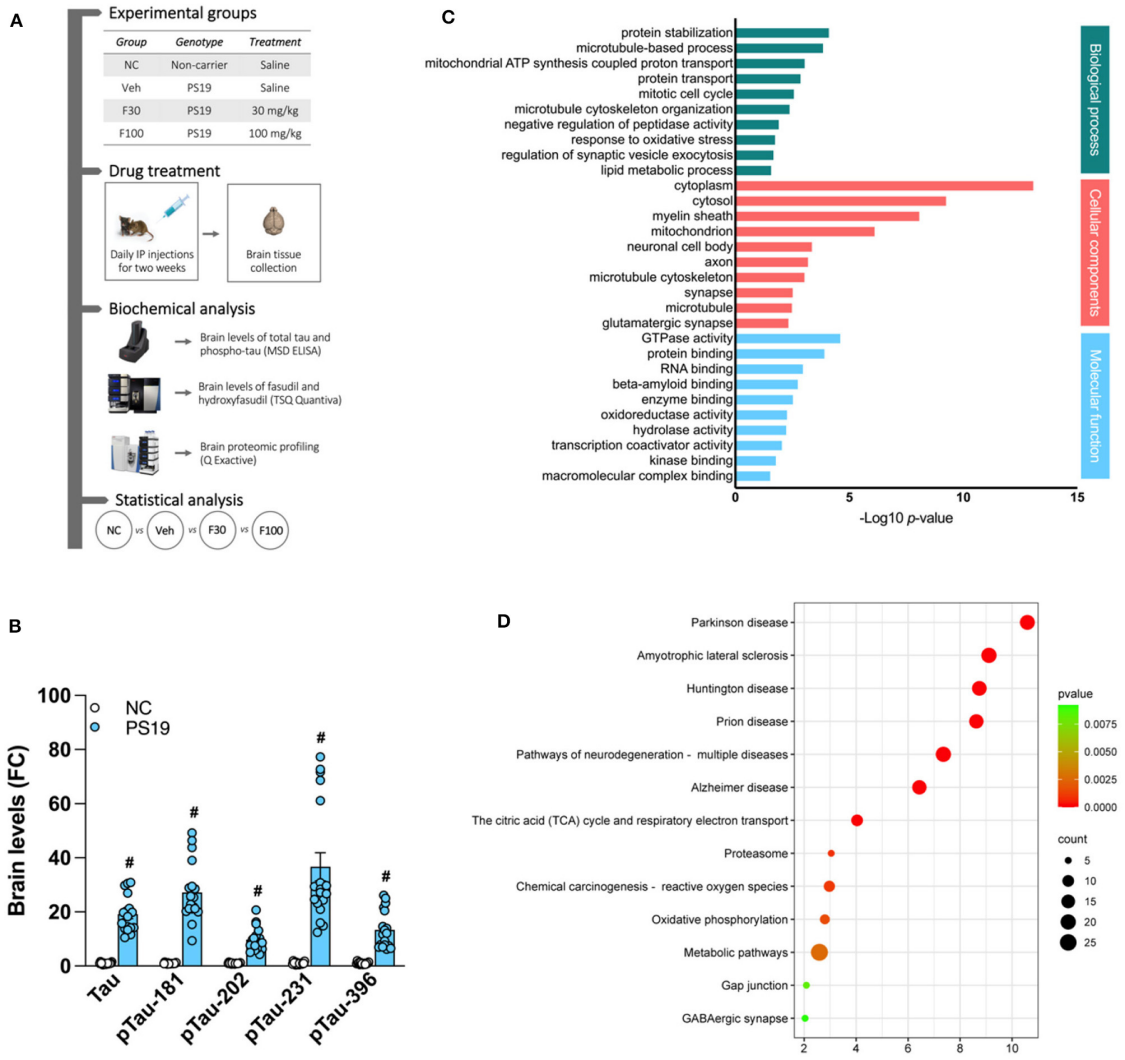
PS19 transgenic mice brain proteomic profile. **(A)** Graphical representation of the experimental timeline of this study including groups, drug treatment, biochemical and statistical analysis performed. **(B)** Levels of total tau (Tau) and phosphorylated tau (pTau)-181, −202, −231, and −396 in the brain of PS19 transgenic mice as compared to non-carrier (NC) mice measured with MSD ELISA. **(C)** Gene ontology enrichment analysis of differentially expressed proteins in the brain of PS19 as compared to NC mice. Significantly enriched biological process, cellular components, and molecular functions are represented and were obtained with DAVID. **(D)** KEGG and Reactome pathway enrichment analysis of differentially expressed proteins in the brain of PS19 as compared to NC mice. Bubble plot represents the top significant pathways.

The publisher apologizes for this mistake. The original article has been updated.

