# Supplementary material for: Effect of the ROCK inhibitor fasudil on the brain proteomic profile in the tau transgenic mouse model of Alzheimer's disease
Source: Front Aging Neurosci. 2024 Feb 19;16:1323563. doi: 10.3389/fnagi.2024.1323563 (PMC10911083; doi:10.3389/fnagi.2024.1323563)
Supplement: Supplementary file 1 [file Image_1.pdf]

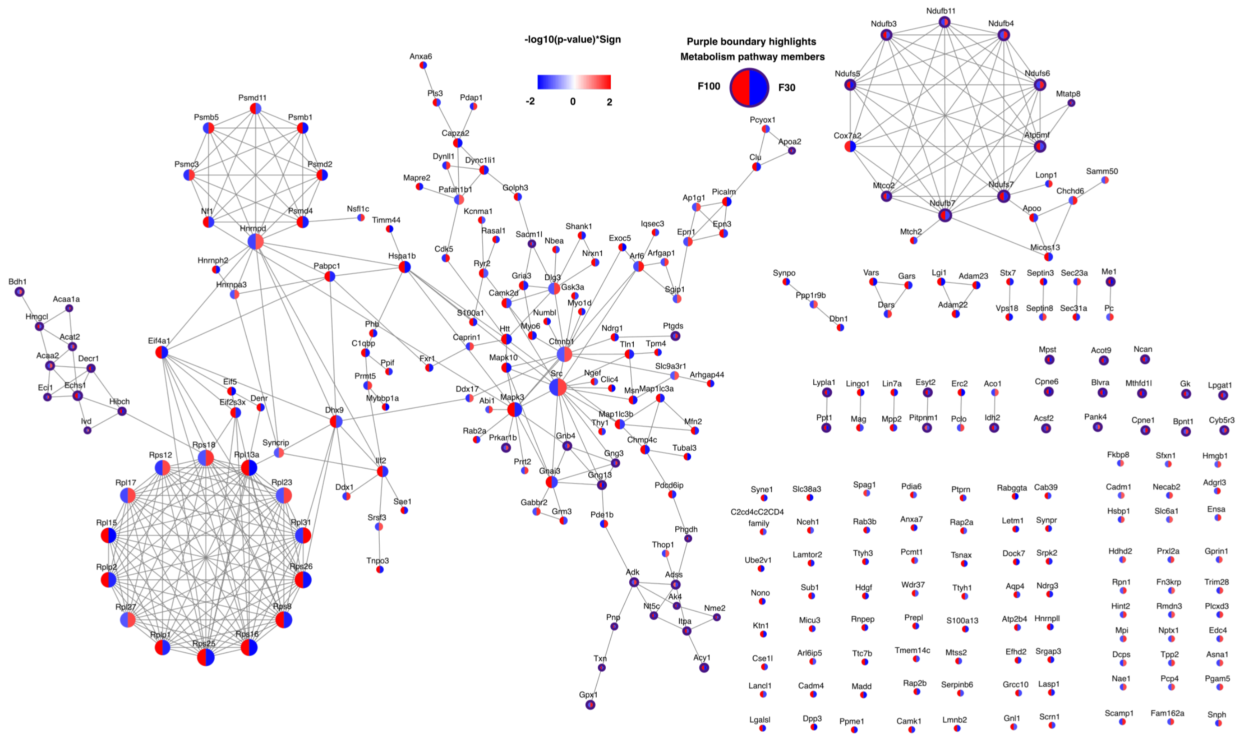

Supplementary figure 1. Network of proteins with significant changes upon the treatment of fasudil at two dosages. A total of 283 proteins showing significant changes upon both treatments, while all of them showing opposite direction of changes in F100 vs Veh and F30 vs Veh, as it combines the members from both “F100 Up - F30 Down” and “F100 Down - F30 Up” groups. Specifically, 188 members of the F100 Up - F30 Down group and 95 from the F30 Up - F100 Down group. Each node represents one protein and 182/283 nodes were connected through 380 high confidence PPIs. Each node face was divided into two halves. The sector on the left was colored based on -log10 transformation of p-value from Tukey’s test for F100 vs. Veh, while the sector on the right was colored according to F30 vs. Veh. P-values corresponding to up-regulation after treatment were red, while blue represented down-regulation after treatment ($p \geq 0.05$ was represented in white). Each edge represents one PPI record with combined confidence score ≥ 0.7 from STRING database.
